# Nonlinear Predictive Motion Control for Autonomous Mobile Robots Considering Active Fault-Tolerant Control and Regenerative Braking

**DOI:** 10.3390/s22103939

**Published:** 2022-05-23

**Authors:** Peng Hang, Baichuan Lou, Chen Lv

**Affiliations:** School of Mechanical and Aerospace Engineering, Nanyang Technological University, Singapore 639798, Singapore; peng.hang@ntu.edu.sg (P.H.); baichuan.lou@ntu.edu.sg (B.L.)

**Keywords:** autonomous mobile robot, motion control, nonlinear model predictive control, active fault-tolerant control, regenerative braking

## Abstract

To further advance the performance and safety of autonomous mobile robots (AMRs), an integrated chassis control framework is proposed. In the longitudinal motion control module, a velocity-tracking controller was designed with the integrated feedforward and feedback control algorithm. Besides, the nonlinear model predictive control (NMPC) method was applied to the four-wheel steering (4WS) path-tracking controller design. To deal with the failure of key actuators, an active fault-tolerant control (AFTC) algorithm was designed by reallocating the driving or braking torques of the remaining normal actuators, and the weighted least squares (WLS) method was used for torque reallocation. The simulation results show that AMRs can advance driving stability and braking safety in the braking failure condition with the utilization of AFTC and recapture the braking energy during decelerations.

## 1. Introduction

Compared with the traditional automated guided vehicles (AGVs), autonomous mobile robots (AMRs) have higher flexibility and intelligence, representing a more sophisticated, flexible, and cost-effective technology, in favor of smart manufacturing, smart factory, and intelligent logistics [1]. AMRs are usually equipped with multiple actuators for steering, drive and brake. Therefore, AMR is an over-actuated system since each wheel can provide independent traction force [2]. It is a critical issue to realize the coordinated control between multiple actuators [3,4].

In recent years, different kinds of advance control methods have been applied to the motion control of robots, including optimal control [5], model predictive control (MPC) [6], Reinforcement Learning (RL)-based control approach [7], adaptive neural network [8], and neuroadaptive learning algorithms [9]. The chassis control of AMR usually consists of longitudinal motion control and lateral motion control [10]. Longitudinal motion control is associated with the drive and brake actuators, e.g., in-wheel motors (IWMs) and electro-mechanical brake (EMB) systems. In longitudinal motion control of AMRs, velocity-tracking control is in favor of the autonomous driving [11]. In [12], a parameter-varying controller was designed for velocity tracking, which showed high robustness. In [13], the MPC method was used for velocity-tracking controller design, which could recover the braking energy with brake torque allocation. In [14], an adaptive sliding mode control (ASMC) algorithm using Radial Basis Function (RBF) neural network was applied to the velocity-tracking controller design, which could deal with external disturbances. Besides, Antilock Braking System (ABS), Acceleration Slip Regulation (ASR), and traction control have also been widely studied in longitudinal motion control for AMRs [15,16,17]. In the lateral motion control of AMRs, path-tracking is the main task for autonomous driving [18]. In [19], a linear quadratic regulator (LQR) technique was used for the four-wheel steering (4WS) path-tracking controller design. However, it showed poor robustness in dealing with uncertainties and disturbances. To reduce the effect of uncertainties in vehicle parameters, a robust path-tracking controller was designed with a μ-synthesis approach [20]. The MPC approach has been widely used in the path-tracking control of AMRs [21]. In [22], an adaptive path-tracking strategy was proposed based on MPC and fuzzy rules, which could guarantee vehicle stability under high-speed and large-curvature conditions. In [23], a Tube-based MPC method was applied to the path-tracking controller design, which showed strong robustness to address uncertainties and disturbances. In [24], an iterative learning control (ILC) method was used for the path-tracking control of AMR, which could improve the path-tracking performance significantly.

To deal with the failure of actuators, a fault-tolerant control has been widely studied [25,26,27]. In [28], a synthesis method was applied to the reconfigurable fault-tolerant control system, which could deal with the failure of steering actuators. With the driving force allocation control method, the vehicle can reconstruct the distribution control strategy on-line under fault conditions, realizing active fault tolerance [29]. In [30], the linear-quadratic control method and the control Lyapunov function technique were used to design the hybrid fault-tolerant control algorithm for the four-wheel-driving vehicle, which can address the actuator failure in the path-tracking process. In [31], a robust fault-tolerant control scheme was designed for distributed actuated electric vehicles, which integrated cooperative game and terminal sliding mode control (SMC) into the framework of the feedback linearization method (FLM). In [32], a fault tolerant sliding mode predictive control (SMPC) strategy was proposed to address the actuator failure, in which SMC was used to improve the robustness of the MPC in the presence of modeling uncertainties and disturbances. In [33], a novel quantized SMC strategy based on switching mechanism was proposed to compensate for actuator failure effects. In [34], the minimax MPC in the delta-domain was deployed to achieve the tracking performance under the actuator fault, system uncertainties, and disturbance.

Most studies only consider the failure of one actuator, which cannot cover all failure conditions. In this research, all kinds of failure conditions of IWMs were studied. Besides, few studies consider the regenerative braking and actuator failure in the motion control process of AMR at the same time. The contributions of this research are summarized as follow: (1) To deal with the system nonlinearity and external disturbances, an integrated feedforward and feedback control algorithm was designed for longitudinal motion control of AMR; (2) To realize the collaborative steering of 4WS, the nonlinear model predictive control (NMPC) method was applied to the path-tracking controller design; (3) To address the braking failure of actuators, an active fault-tolerant control (AFTC) algorithm was designed for AMR by redistributing the braking torques of the rest normal actuators.

The rest of this paper is organized as follows. Section 2 gives the problem description and control framework for AMR. The modelling work for control algorithm design is described in Section 3. Section 4 presents the control algorithm design for AMR. Then, the simulation tests are described in Section 5. Finally, Section 6 provide some conclusions and suggests future work.

## 2. Problem Description and Control Framework

### 2.1. Control Problem Description for AMR

To realize autonomous driving, the motion control for AMR mainly consists of longitudinal motion control and lateral motion control. Lateral motion control is reflected by the path-tracking issue. Longitudinal motion control is related to the drive and brake control, which is a critical issue in this study.

IWMs are the key components for AMR. On one hand, in-wheel motors can be used to drive the AMR. On the other hand, regenerative braking can be realized with in-wheel motors, recovering the braking energy. AMR is usually equipped with four in-wheel motors for independent drive, and four EMB systems for independent braking. Due to so many actuators, the reliability of the system is decreased. Therefore, safety is a critical issue for AMR. In the braking process, if braking failure of actuators occurs, this reduces safety. To maximize regenerative braking energy, IWMs have higher braking priority than EMBs. EMBs are usually used to compensate the rest braking force. Therefore, we mainly discuss the braking failure of IWMs in this paper.

**Figure 1:**
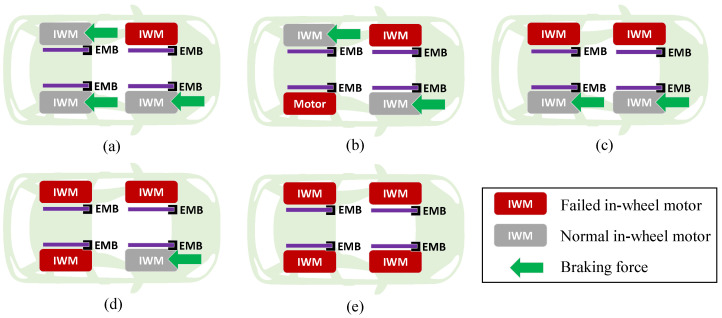
Braking failure of in-wheel motors: (**a**) Failure of one in-wheel motor; (**b**) failure of two in-wheel motors on two sides; (**c**) failure of two in-wheel motors on the same side; (**d**) failure of three in-wheel motors; (**e**) failure of four in-wheel motors.

Figure 1 shows the braking failure conditions of IWMs divided into five types, i.e., failure of one IWM, failure of two IWMs on two sides, failure of two IWMs on the same side, failure of three IWMs, and failure of four IWMs. In this paper, the AFTC algorithm is proposed to deal with all kinds of braking failure of IWMs.

### 2.2. Chassis Control Framework for AMR

**Figure 2:**
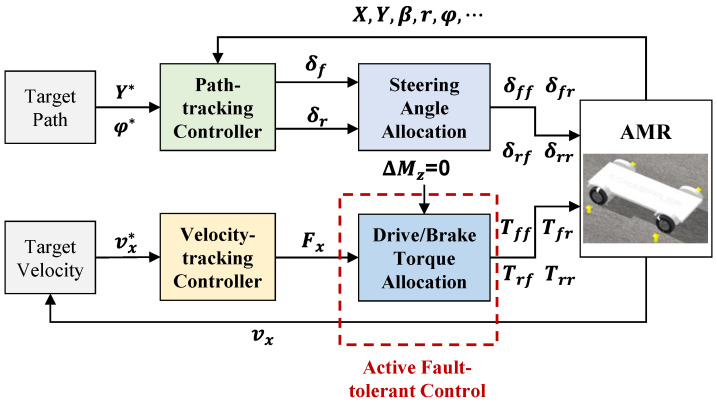
Chassis control framework for AMR.

The chassis control framework for AMR is illustrated in Figure 2, which mainly consists of longitudinal motion control and lateral motion control, i.e., the velocity-tracking control and the path-tracking control. In the path-tracking control module, NMPC is applied to the controller design. Based on the target path and the feedbacked vehicle state, the path-tracking controller outputs the front and rear wheel steering angels. In the velocity-tracking control module, an integrated feedforward and feedback controller is designed. To deal with the braking failure of IWMs, an AFTC module is designed after the velocity-tracking controller. With the torque redistribution of IWMs and EMBs, the AFTC algorithm is able to maximize the regenerative braking energy and guarantee safety at the same time.

## 3. Modelling

### 3.1. Vehicle Dynamic Model

Some assumptions are made in this paper. First, only seven degrees of freedom are considered for the vehicle dynamic model, i.e., longitudinal motion, lateral motion, yaw motion of the vehicle and the four wheels’ motion. Pitch motion, roll motion, and vertical motion of AMR are ignored. Drive anti-skid control is not considered in the longitudinal motion control strategy. This paper mainly focuses on the velocity-tracking control and braking control. Additionally, the longitudinal acceleration of the wheel center is considered equal to the longitudinal acceleration of the AMR at CG.

The longitudinal dynamic model is derived as follows [35].

(1) $$m\left({\dot{v}}_{x}-v_{y}r\right)=F_{x}-F_{w}-F_{f}$$

(2) $$F_{x}=F_{xfl}\cos\delta_{fl}+F_{xfr}\cos\delta_{fr}+F_{xrl}\cos\delta_{rl}+F_{xrr}\cos\delta_{rr}$$

(3) $$F_{w}=C_{D}A\rho v_{x}^{2}/2$$

(4) $$F_{f}=f_{r}mg$$

where $v_{x}$ and $v_{y}$ denote the longitudinal and lateral velocities, *r* denotes the yaw rate at the center of gravity (CG), $F_{x}$ denotes the total longitudinal tire force acting on the vehicle. $F_{w}$ and $F_{f}$ denote the wind resistance and the rolling resistance, respectively. m denotes the vehicle mass, $\delta_{i}$ (*i* = *fl*, *fr*, *rl*, *rr*) denotes the steering angle of each wheel (*fl* denotes the front left wheel, *fr* denotes the front right wheel, *rl* denotes the rear left wheel, and *rr* denotes the rear right wheel). $F_{xi}$ (*i* = *fl*, *fr*, *rl*, *rr*) denotes the longitudinal force of each tire, $C_{D}$, A and ρ denote the air resistance coefficient, windward area and air density, respectively., and $f_{r}$ and g denote the rolling resistance coefficient and the gravitational acceleration.

The lateral dynamic model is expressed by [36]

(5) $$m\left({\dot{v}}_{y}+v_{x}r\right)=F_{y}$$

(6) $$F_{y}=F_{yfl}\cos\delta_{fl}+F_{yfr}\cos\delta_{fr}+F_{yrl}\cos\delta_{rl}+F_{yrr}\cos\delta_{rr}$$

where $F_{y}$ denotes the total lateral tire force acting on the vehicle. $F_{yi}$ (*i* = *fl*, *fr*, *rl*, *rr*) denotes the lateral force of each tire, which is expressed with the Dugoff tire model [37].

The yaw dynamic model is written according to [38]

(7) $$I_{z}\dot{r}=M_{z}$$

(8) $$M_{z}=\left(F_{yfl}\cos\delta_{fl}+F_{yfr}\cos\delta_{fr}\right)l_{f}-\left(F_{yrl}\cos\delta_{rl}+F_{yrr}\cos\delta_{rr}\right)l_{r}+\Delta M_{z}$$

where $M_{z}$ denotes the total yaw moment acting on the vehicle, $I_{z}$ denotes the yaw inertia moment, $l_{f}$ denotes the distance from the front axle to CG, and $l_{r}$ denotes the distance from the rear axle to CG. $\Delta M_{z}$ is the external yaw moment, which is created by the torque difference between left and right wheels.

(9) $$\Delta M_{z}=[-F_{xfl}\cos\delta_{fl}+F_{xfr}\cos\delta_{fr}-F_{xrl}\cos\delta_{rl}+F_{xrr}\cos\delta_{rr}]\frac{B}{2}$$

where B denotes the vehicle track. 

Additionally, the dynamic model of each wheel is derived by

(10) $$I_{w}{\dot{\omega }}_{i}=T_{i}-F_{xi}R_{w}$$

where $T_{i}$ denotes the wheel torque, $T_{i}=T_{di}-T_{bi}$, $T_{di}$ and $T_{bi}$ denote the drive and brake torques, respectively, $\omega_{i}$ and $R_{w}$ denote the angular velocity of each wheel and the rolling radius of the tire, respectively, and $I_{w}$ denotes the wheel moment of inertia.

### 3.2. Path-Tracking Model

**Figure 3:**
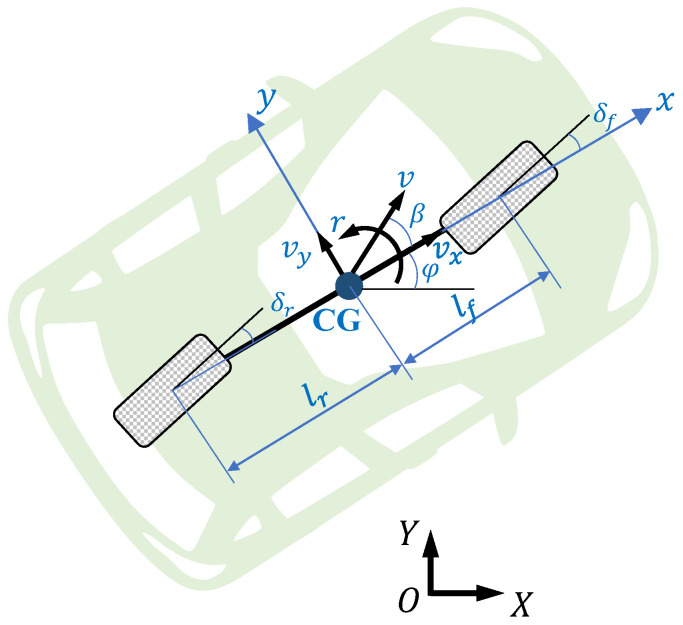
Single-track vehicle model.

As Figure 3 shows, the 4-wheel vehicle model is usually simplified to be a single-track model to simplify the controller design [39]. The steering angle transformation relationship between the two models follows the Ackerman steering geometry [40].

(11) $$\begin{array}{cc} \tan\delta_{fl}=\frac{\tan\delta_{f}}{1-\frac{B}{2l}\left(\tan\delta_{f}-\tan\delta_{r}\right)}, & \tan\delta_{fr}=\frac{\tan\delta_{f}}{1+\frac{B}{2l}\left(\tan\delta_{f}-\tan\delta_{r}\right)}\\ \tan\delta_{rl}=\frac{\tan\delta_{r}}{1-\frac{B}{2l}\left(\tan\delta_{f}-\tan\delta_{r}\right)}, & \tan\delta_{rr}=\frac{\tan\delta_{r}}{1+\frac{B}{2l}\left(\tan\delta_{f}-\tan\delta_{r}\right)} \end{array}$$

where $\delta_{f}$ and $\delta_{r}$ denote the front and rear steering angles, and l denotes the distance from the front axle to the rear axle.

The yaw angel φ and lateral position Y of AMR at CG are expressed as

(12) $$\{\begin{array}{c} \dot{\varphi }=r\\ \dot{Y}=v_{x}\sin\varphi +v_{y}\cos\varphi \end{array}$$

A combination of (5), (7), (11) and (12) yields the following path-tracking model for AMR.

(13) $$\begin{array}{c} \dot{x}\left(t\right)=f\left(x\left(t\right),u\left(t\right)\right)\\ y\left(t\right)=g\left(x\left(t\right),u\left(t\right)\right) \end{array}$$

(14) $$f\left(x\left(t\right),u\left(t\right)\right)=\left[\begin{array}{c} -v_{x}r+\frac{\sum F_{y}}{m}\\ \frac{\sum M_{z}}{I_{z}}\\ r\\ v_{x}\sin\varphi +v_{y}\cos\varphi \end{array}\right]$$

(15) $$g\left(x\left(t\right),u\left(t\right)\right)=\left[\begin{array}{cccc} 0 & 0 & 1 & 0\\ 0 & 0 & 0 & 1 \end{array}\right]x\left(t\right)$$

where the state vector $x={\left[v_{y},\;r,\;\varphi ,\;Y\right]}^{T}$, the output vector $y={\left[\;\varphi ,\;Y\right]}^{T}$, and the control vector $u={\left[\delta_{f},\delta_{r}\right]}^{T}$.

## 4. Control Algorithm Design

### 4.1. Velocity-Tracking Control Algorithm

For velocity-tracking controller design, (1) is rewritten as follows.

(16) $$m{\dot{v}}_{x}=F_{x}-F_{w}-F_{f}+F_{c}$$

where $F_{c}=mv_{y}r$.

After Taylor expansion of (2) regarding $\cos\delta_{i}:$

(17) $$F_{x}=F_{xfl}+F_{xfr}+F_{xrl}+F_{xrr}+F_{d}$$

where higher-order terms are placed in $F_{d}$.

Then, the simplified longitudinal dynamic model can be expressed as

(18) $$m{\dot{v}}_{x}=F_{xfl}+F_{xfr}+F_{xrl}+F_{xrr}-F_{w}-F_{f}+F_{c}+F_{d}$$

Based on the wheel dynamic model (10), it can be derived that

(19) $$F_{xi}=\frac{T_{i}-I_{wi}{\dot{v}}_{xi}/R_{w}}{R_{w}}\;\;\;\left(i=fl,fr,rl,rr\right)$$

Substitution of (19) into (18) yields

(20) $$\left(m+\frac{\sum I_{wi}}{R_{w}^{2}}\right){\dot{v}}_{x}=\frac{\sum T_{i}}{R_{w}}-F_{w}-F_{f}+F_{c}+F_{d}$$

The total torque of four wheels $\sum T_{i}$ is defined as the longitudinal control vector, which is made up of the feedforward and feedback controllers, i.e.,

(21) $$\sum T_{i}=u_{ff}+u_{fb}$$

According to the model (20), the feedforward controller is derived as follows.

(22) $$u_{ff}=\left(mR_{w}+\frac{\sum I_{wi}}{R_{w}}\right){\dot{v}}_{x}^{*}+(F_{w}+F_{f}-F_{c})R_{w}$$

where $v_{x}^{*}$ denotes the target velocity. The feedforward controller is mainly used to compensate the control error caused by the nonlinearity of the system.

Substitution of (22) into (20) yields

(23) $$\left(m+\frac{\sum I_{wi}}{R_{w}^{2}}\right){\dot{e}}_{v_{x}}=\frac{u_{fb}}{R_{w}}+F_{d}$$

where $e_{v_{x}}$ denotes the velocity tracking error, i.e., $e_{v_{x}}=v_{x}-v_{x}^{*}$.

The feedback controller is designed by PID. Furthermore, defining the state vector $x_{l}={\left[\int_{0}^{t}e_{v_{x}}d\tau ,\;e_{v_{x}},{\dot{e}}_{v_{x}}\right]}^{T}$, control vector $u_{l}={\left[u_{fb},{\dot{u}}_{fb}\right]}^{T}$, disturbance vector $d_{l}={\left[F_{d},{\dot{F}}_{d}\right]}^{T}$, then, (23) can be written in the state-space form.

(24) $$\begin{array}{l} \dot{x_{l}}=A_{l}x_{l}+B_{l}u_{l}+E_{l}d_{l}\\ y_{l}=C_{l}x_{l} \end{array}$$

where $A_{l}=\left[\begin{array}{ccc} 0 & 1 & 0\\ 0 & 0 & 0\\ 0 & 0 & 0 \end{array}\right]$, $B_{l}=\left[\begin{array}{cc} 0 & 0\\ B_{s} & 0\\ 0 & B_{s} \end{array}\right]$, $C_{l}=\left[\begin{array}{ccc} 0 & 1 & 0 \end{array}\right]$, $E_{l}=\left[\begin{array}{cc} 0 & 0\\ E_{s} & 0\\ 0 & E_{s} \end{array}\right]$, $B_{s}=\frac{R_{w}}{mR_{w}^{2}+\sum I_{wi}}$, and $E_{s}=\frac{R_{w}^{2}}{mR_{w}^{2}+\sum I_{wi}}$.

To solve the feedback PID controller, the following performance index function is constructed:

(25) $$J_{PID}=\int_{0}^{\infty }\left(y_{l}^{T}Q_{l}y_{l}+u_{l}^{T}R_{l}u_{l}\right)dt$$

where $Q_{l}$ and $R_{l}$ are weighting matrix, $Q_{l}=10^{3}$, $R_{l}=I_{2\times 2}$.

Furthermore, the solution problem of the feedback PID controller can be transformed into the minimization of the performance index function, i.e.,

(26) $$\min\;J_{PID}\left(K\right)$$

Finally, the linear-quadratic optimization approach is used to solve the feedback PID controller [41,42].

### 4.2. Path-Tracking Control Algorithm

For the path-tracking controller design, the path-tracking model (13) is expressed in the discreate state-space form as follows.

(27) $$\begin{array}{l} x\left(k+1\right)=F\left(x\left(k\right),u\left(k\right)\right)\\ y\left(k\right)=G\left(x\left(k\right),u\left(k\right)\right) \end{array}$$

where F(x(k),u(k))=x(k)+Tf(x(k),u(k)), G(x(k),u(k))=g(x(k),u(k)), T denotes the sampling time, and T=0.02s.

Based on the discreate model (27), NMPC is applied to the path-tracking controller design. The prediction horizon and the control horizon are defined by $N_{p}$ and $N_{c}$, $N_{p}\geq N_{c}$. $N_{p}=10,$ and $N_{c}=5$. Then, the predictive outputs are derived as follows.

(28) $$\begin{array}{c} y\left(k+1\right)=G\left(x\left(k+1\right),u\left(k+1\right)\right)\\ y\left(k+2\right)=G\left(x\left(k+2\right),u\left(k+2\right)\right)\\ \vdots \\ y\left(k+N_{c}\right)=G\left(x\left(k+N_{c}\right),u\left(k+N_{c}\right)\right)\\ y\left(k+N_{c}+1\right)=G\left(x\left(k+N_{c}+1\right),u\left(k+N_{c}\right)\right)\\ \vdots \\ y\left(k+N_{p}\right)=G\left(x\left(k+N_{p}\right),u\left(k+N_{c}\right)\right) \end{array}$$

Based on (28), this yields the output sequence as follows.

(29) $$y\left(k+1\right)={\left[y\left(k+1\right),y\left(k+2\right),\cdots ,y\left(k+N_{p}\right)\right]}^{T}$$

Besides, the reference output sequence is expressed by

(30) $$\hat{y}\left(k+1\right)={\left[\hat{y}\left(k+1\right),\hat{y}\left(k+2\right),\cdots ,\hat{y}\left(k+N_{p}\right)\right]}^{T}$$

where $\hat{y}\left(k+p\right)={\left[\varphi^{*}\left(k+p\right),\;Y^{*}\left(k+p\right)\right]}^{T},\;p=1,\cdots ,N_{p}$, $\varphi^{*}\left(k+p\right)$ and $Y^{*}\left(k+p\right)$ denote the reference values of yaw angle and lateral position.

Moreover, the control sequence is expressed as follows.

(31) $$u\left(k+1\right)={\left[u\left(k+1\right),u\left(k+2\right),\cdots ,u\left(k+N_{c}\right)\right]}^{T}$$

The proposed path tracking controller aims to minimize the tracking error ${\left\|y\left(k+1\right)-\hat{y}\left(k+1\right)\right\|}_{2}$ with the smallest control energy ${\left\|u(k+1)\right\|}_{2}$. Furthermore, the following cost function is constructed.

(32) $$\begin{array}{c} J\left(k\right)=\sum_{i=1}^{N_{p}}{\left[y\left(k+i|k\right)-\hat{y}\left(k+i|k\right)\right]}^{T}Q\left[y\left(k+i|k\right)-\hat{y}\left(k+i|k\right)\right]\\ +\sum_{i=0}^{N_{c}-1}{\left[u\left(k+i|k\right)\right]}^{T}R\left[u\left(k+i|k\right)\right] \end{array}$$

where Q and R are diagonal weighting matrices, $Q=\mathrm{diag}\left\{8\times 10^{3},\;10^{4}\right\}$, $R=\mathrm{diag}\left\{5\times 10^{5},\;10^{6}\right\}$.

Finally, the NMPC path-tracking controller can be solved with the following optimization.

(33) $$\begin{array}{l} \underset{u\left(k\right)}{\min}J\left(k\right)\\ s.t.\\ x\left(k+i|k\right)=F\left(x\left(k+i-1|k\right),u\left(k+i-1|k\right)\right)\\ u_{\min}\leq u\left(k+i|k\right)\leq u_{\max} \end{array}$$

### 4.3. Active Fault-Tolerant Control Algorithm

In this section, we only discuss the braking failure of IWMs. If IWMs have failure in the driving process, the AFTC mechanism is triggered immediately. After that, the AMR starts braking to guarantee safety. Therefore, we do not discuss the driving failure of IWMs independently.

Since the total torque of four wheels $\sum T_{i}$ has been worked out based on Section 4.1., it yields that

(34) $$\sum T_{i}=T^{IWMs}+T^{EMBs}$$

where $T^{IWMs}$ and $T^{EMBs}$ denote the total torques of four IWMs and four EMBs, respectively, i.e., $T^{IWMs}=T_{fl}^{IWM}+T_{fr}^{IWM}+T_{rl}^{IWM}+T_{rr}^{IWM}$, $T^{EMBs}=T_{fl}^{EMB}+T_{fr}^{EMB}+T_{rl}^{EMB}+T_{rr}^{EMB}$.

Besides, the external yaw moment is generated by IWMs and EMBs, i.e.,

(35) $$\Delta M_{z}=\Delta M_{z}^{IWMs}+\Delta M_{z}^{EMBs}$$

where $\Delta M_{z}^{IWMs}$ and $\Delta M_{z}^{EMBs}$ denote the external yaw moment generated by IWMs and EMBs, respectively.

**Figure 4:**
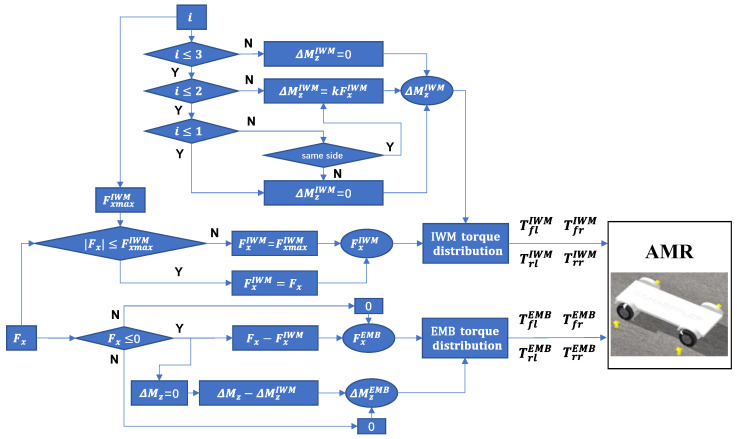
AFTC flowchart for all kinds of braking failure of IWMs.

To guarantee yaw stability, $\Delta M_{z}=0$. The following work aims to distribute the torque for each IWM and EMB based on (34) and (35). Figure 4 shows the AFTC flowchart to deal with all kinds of braking failure of IWMs.

To maximize the regenerative braking energy, IWMs has higher braking priority than EMBs. Therefore, the first step is to determine if $|F_{x}|\leq F_{xmax}^{IWM}$, $F_{xmax}^{IWM}$ denotes the braking force boundaries of all normal IWMs, which is related to the failure number of IWM, i.e., *i* in Figure 4. If $|F_{x}|\leq F_{xmax}^{IWM}$, $F_{x}^{IWM}=F_{x}$, else $F_{x}^{IWM}=F_{xmax}^{IWM}$ and EMBs will compensate the rest braking force, i.e., $F_{x}^{EMB}=F_{x}-F_{x}^{IWM}$, where $F_{x}^{IWM}$ and $F_{x}^{EMB}$ denote the total braking force of four IWMs and four EMBs.

Since $\Delta M_{z}^{IWM}$ cannot be zero under some failure conditions, e.g., failure of two IWMs on the same side and failure of three IWMs, the generated $-\Delta M_{z}^{IWM}$ will be compensated by $\Delta M_{z}^{EMB}$.

Once $F_{x}^{IWM}$, $F_{x}^{EMB}$, $\Delta M_{z}^{IWM}$ and $\Delta M_{z}^{EMB}$ are determined, the torque distribution algorithm will work to work out $T_{fl}^{IWM}$, $T_{fr}^{IWM}$, $T_{rl}^{IWM}$, $T_{rr}^{IWM}$, $T_{fl}^{EMB}$, $T_{fr}^{EMB}$, $T_{rl}^{EMB}$, $T_{rr}^{EMB}$. $F_{x}^{IWM}$ and $F_{x}^{EMB}$ can be derived from $T^{IWM}$ and $T^{EMB}$ based on (19).

For IWMs, the following torque distribution model is derived.

(36) $$\Lambda^{IWM}=\eta^{IWM}\Theta^{IWM}$$

(37) $$\eta^{IWM}=\left[\begin{array}{cccc} 1 & 1 & 1 & 1\\ -\frac{B}{R_{w}} & \frac{B}{R_{w}} & -\frac{B}{R_{w}} & \frac{B}{R_{w}} \end{array}\right]\lambda$$

(38) $$\lambda =\left[\begin{array}{cccc} \lambda_{fl} & 0 & 0 & 0\\ 0 & \lambda_{fr} & 0 & 0\\ 0 & 0 & \lambda_{rl} & 0\\ 0 & 0 & 0 & \lambda_{rr} \end{array}\right]$$

(39) $$\lambda_{i}=\{\begin{array}{l} 1,\;\;\;\mathrm{normal}\\ 0,\;\;{\;\mathrm{failure}\;\mathrm{of}\;\mathrm{IWM}}_{i} \end{array}\left(i=fl,fr,\;rl,\;rr\right)$$

where $\Lambda^{IWM}={\left[T^{IWM},\Delta M_{z}^{IWM}\right]}^{T}$ and $\Theta^{IWM}={\left[T_{fl}^{IWM},T_{fr}^{IWM},T_{rl}^{IWM},T_{rr}^{IWM}\right]}^{T}$.

**Figure 5:**
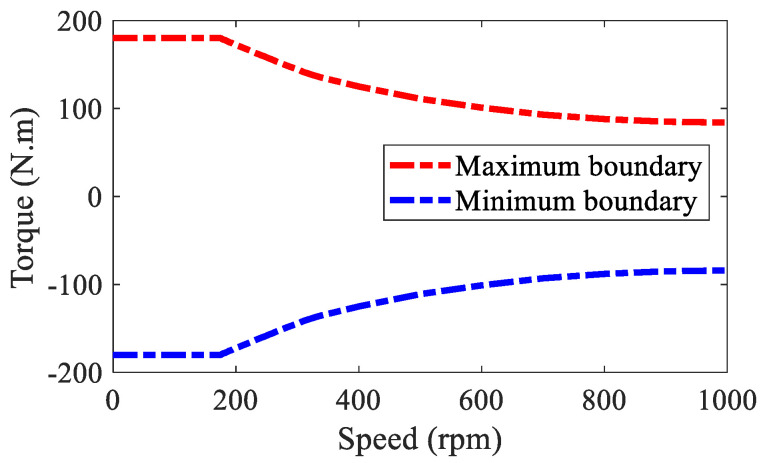
Control boundaries of IWM.

Based on (36), the weighted least squares (WLS) method is used to distribute the torques of IWMs. The cost function for IWM torque distribution is constructed as follows.

(40) $$\begin{array}{l} \Psi^{IWM}=\rho^{IWM}{\left\|\omega_{\Lambda }^{IWM}\left(\eta^{IWM}\Theta^{IWM}-\Lambda^{IWM}\right)\right\|}_{2}^{2}+{\left\|\omega_{\Theta }^{IWM}\left(\Theta^{IWM}-\Theta_{d}^{IWM}\right)\right\|}_{2}^{2}\\ s.t.\;\;\Theta_{\min}^{IWM}\leq \Theta^{IWM}\leq \Theta_{\max}^{IWM} \end{array}$$

where $\rho^{IWM}$ denotes the weighting coefficient, which is usually set very large to minimize the torque distribution error, $\rho^{IWM}=10^{6}$. $\Theta_{d}^{IWM}$ denotes the desired control vector, $\Theta_{d}^{IWM}={\left[0,\;0,\;0,\;0\right]}^{T}$. $\Theta_{\min}^{IWM}$ and $\Theta_{\max}^{IWM}$ denote the minimum and maximum control boundaries of $\Theta^{IWM}$, which is shown in Figure 5. $\omega_{\Lambda }^{IWM}$ and $\omega_{\Theta }^{IWM}$ denote the weighting matrices. In this paper, $T^{IWM}$ and $\Delta M_{z}^{IWM}$ have the same allocation weights, i.e., $\omega_{\Lambda }^{IWM}=\mathrm{diag}\;\left[1,1\right]$, $T_{i}^{IWM}\;\left(i=fl,\;fr,\;rl,\;rr\right)$ and $F_{zi}$ are positively correlated, where $F_{zi}$ denotes the vertical load of each wheel. Thus, $\omega_{\Theta }^{IWM}=\mathrm{diag}\left[\frac{1}{F_{zfl}},\frac{1}{F_{zfr}},\frac{1}{F_{zrl}},\frac{1}{F_{zrr}}\right]$.

Furthermore, (40) is rewritten as

(41) $$\Psi^{IWM}={\|\underset{A^{IWM}}{\underbrace{\left[\begin{array}{c} {\left(\rho^{IWM}\right)}^{1/2}\omega_{\Lambda }^{IWM}\eta^{IWM}\\ \omega_{\Theta }^{IWM} \end{array}\right]}}\Theta^{IWM}-\underset{B^{IWM}}{\underbrace{\left[\begin{array}{c} {\left(\rho^{IWM}\right)}^{1/2}\omega_{\Theta }^{IWM}\Lambda^{IWM}\\ \omega_{\Theta }^{IWM}\Theta_{d}^{IWM} \end{array}\right]}}\|}_{2}^{2}$$

Then, the WLS method for IWMs torque distribution is described as follows.

(42) $$\begin{array}{l} \underset{\Theta^{IWM}}{\min}{\left\|A^{IWM}\Theta^{IWM}-B^{IWM}\right\|}_{2}^{2}\\ s.t.\;\;\Theta_{\min}^{IWM}\leq \Theta^{IWM}\leq \Theta_{\max}^{IWM} \end{array}$$

Based on (42), the torques for four IWMs, i.e., $T_{fl}^{IWM}$, $T_{fr}^{IWM}$, $T_{rl}^{IWM}$, $T_{rr}^{IWM}$, can be worked out.

For EMBs, the following torque distribution model is derived.

(43) $$\Lambda^{EMB}=\eta^{EMB}\Theta^{EMB}$$

(44) $$\eta^{EMB}=\left[\begin{array}{cccc} 1 & 1 & 1 & 1\\ -\frac{B}{R_{w}} & \frac{B}{R_{w}} & -\frac{B}{R_{w}} & \frac{B}{R_{w}} \end{array}\right]$$

where $\Lambda^{EMB}={\left[T^{EMB},\Delta M_{z}^{EMB}\right]}^{T}$ and $\Theta^{EMB}={\left[T_{fl}^{EMB},T_{fr}^{EMB},T_{rl}^{EMB},T_{rr}^{EMB}\right]}^{T}$.

Based on (43), the cost function for EMBs torque distribution is derived as follows.

(45) $$\begin{array}{l} \Psi^{EMB}=\rho^{EMB}{\left\|\omega_{\Lambda }^{EMB}\left(\eta^{EMB}\Theta^{EMB}-\Lambda^{EMB}\right)\right\|}_{2}^{2}+{\|\omega_{\Theta }^{EMB}\left(\Theta^{EMB}-\Theta_{d}^{EMB})\right\|}_{2}^{2}\\ s.t.\;\;\Theta_{\min}^{EMB}\leq \Theta^{EMB}\leq \Theta_{\max}^{EMB} \end{array}$$

where $\rho^{EMB}$ denotes the weighting coefficient, which is usually set very large to minimize the torque distribution error, $\rho^{EMB}=10^{6},$ $\Theta_{d}^{EMB}$ denotes the desired control vector, $\Theta_{d}^{EMB}={\left[0,\;0,\;0,\;0\right]}^{T}$, $\Theta_{\min}^{EMB}$ and $\Theta_{\max}^{EMB}$ denote the minimum and maximum control boundaries of $\Theta^{EMB}$, $\Theta_{\min}^{EMB}=-200,\;\Theta_{\max}^{EMB}=0,$ $\omega_{\Lambda }^{EMB}$ and $\omega_{\Theta }^{EMB}$ denote the weighting matrices, $\omega_{\Lambda }^{EMB}=\mathrm{diag}\;\left[1,1\right]$, $\omega_{\Theta }^{EMB}=\mathrm{diag}\left[\frac{1}{F_{zfl}},\frac{1}{F_{zfr}},\frac{1}{F_{zrl}},\frac{1}{F_{zrr}}\right]$.

Furthermore, (45) is rewritten as

(46) $$\Psi^{EMB}={\|\underset{A^{EMB}}{\underbrace{\left[\begin{array}{c} {\left(\rho^{EMB}\right)}^{1/2}\omega_{\Lambda }^{EMB}\eta^{EMB}\\ \omega_{\Theta }^{EMB} \end{array}\right]}}\Theta^{EMB}-\underset{B^{EMB}}{\underbrace{\left[\begin{array}{c} {\left(\rho^{EMB}\right)}^{1/2}\omega_{\Theta }^{EMB}\Lambda^{EMB}\\ \omega_{\Theta }^{EMB}\Theta_{d}^{EMB} \end{array}\right]}}\|}_{2}^{2}$$

Then, the WLS method for EMB torque distribution is derived as follows.

(47) $$\begin{array}{l} \underset{\Theta^{EMB}}{\min}{\left\|A^{EMB}\Theta^{EMB}-B^{EMB}\right\|}_{2}^{2}\\ s.t.\;\;\Theta_{\min}^{EMB}\leq \Theta^{EMB}\leq \Theta_{\max}^{EMB} \end{array}$$

Based on (47), the torques for four IWMs, i.e., $T_{fl}^{EMB}$, $T_{fr}^{EMB}$, $T_{rl}^{EMB}$, $T_{rr}^{EMB}$, can be worked out.

## 5. Simulation Results and Analysis

**Figure 6:**
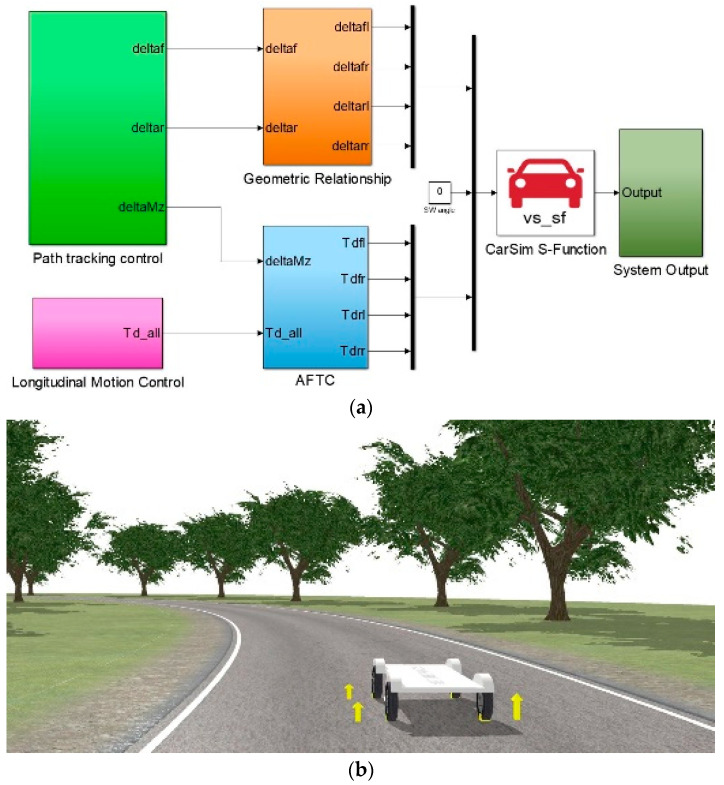
Co-simulation platform based on Carsim and Simulink: (**a**) Control algorithm; (**b**) simulation scenario.

Three simulation cases were designed and carried out via the co-simulation platform based on Carsim and Simulink as shown in Figure 6. Figure 6a shows the Simulink algorithm structure in the co-simulation platform, including the path-tracking control algorithm, longitudinal velocity-tracking control algorithm and the AFTC algorithm. All the control algorithms were carried out in the Simulink software. The real AMR model was built in Carsim software. With the co-simulation of Carsim and Simulink, the effectiveness and feasibility of the proposed algorithm were verified. Figure 6b shows the simulation scenario in Carsim.

### 5.1. Simulation Case 1

In this case, a straight-line braking condition was carried out. The AMR accelerated to 15 m/s and then started to brake after the 10th second. Three kinds of braking modes were compared in this case, i.e., regenerative braking (IWM), mechanical braking (EMB) and hybrid braking (IWM + EMB). The three kinds of braking modes were realized based on the same AMR with the parameters in Table 1 and the same simulation platform in Figure 6. The same velocity-tracking control algorithm and path-tracking control algorithm were utilized. In this case, braking failure was not considered.

**Figure 7:**
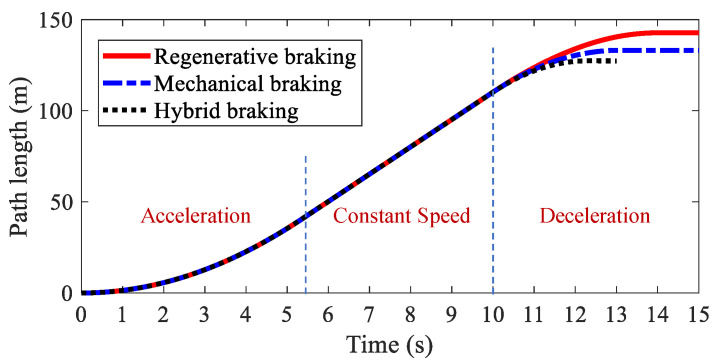
Path length of AMR in Case 1.

**Figure 8:**
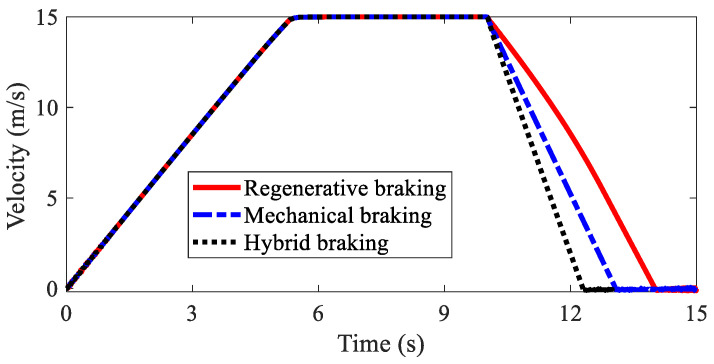
Velocity of AMR in Case 1.

The path lengths of AMR with different kinds of braking modes are illustrated in Figure 7. It was found that regenerative braking had the longest braking distance. The second was mechanical braking, and the shortest was hybrid braking. A detailed analysis is shown in Table 1. The braking distances for the three kinds of braking modes were 42.74 m, 33.15 m, 27.33 m, respectively, and the braking times for the three kinds of braking modes were 4.04 s, 3.09 s, 2.32 s, respectively. Figure 8 shows the velocities of AMR with different kinds of braking modes. Hybrid braking showed the largest deceleration among the three kinds of braking modes. It can be concluded that hybrid braking can shorten the braking distance and braking time remarkably, improving braking safety.

**Figure 9:**
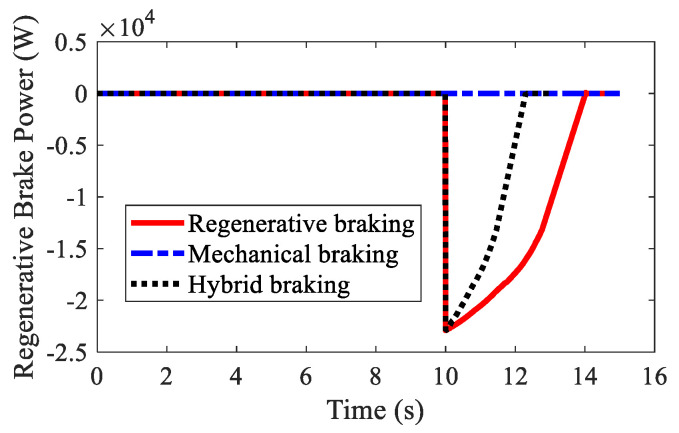
Regenerative braking power of AMR in Case 1.

Regenerative braking powers with different kinds of braking modes are depicted in Figure 9. Mechanical braking cannot recover braking energy. Regenerative braking has larger regenerative braking power than hybrid braking. As shown in Table 2 regenerative braking energies for regenerative braking, mechanical braking, and hybrid braking were $6.10\times 10^{4}$ J, 0 J, and $3.29\times 10^{4}$ J, respectively. Due to the application of EMB in hybrid braking, the hybrid braking mode had smaller regenerative braking energy than the regenerative braking mode.

**Figure 10:**
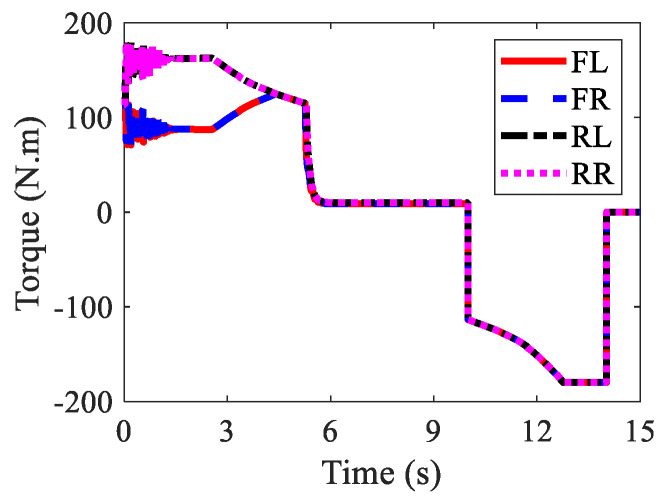
Wheel torques of AMR with regenerative braking in Case 1.

**Figure 11:**
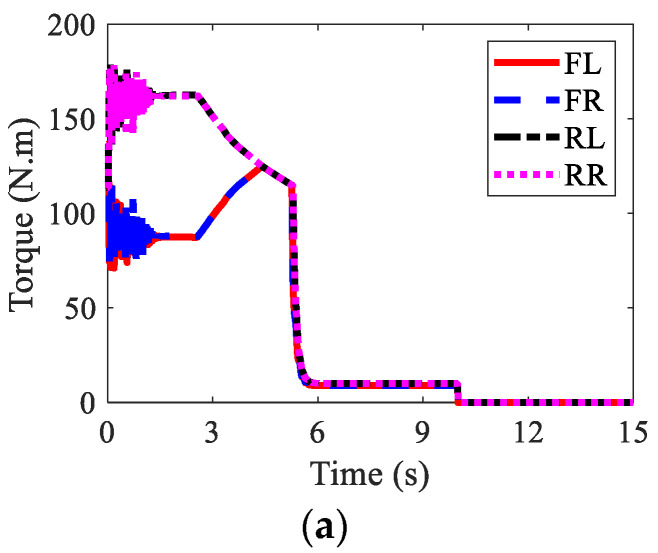

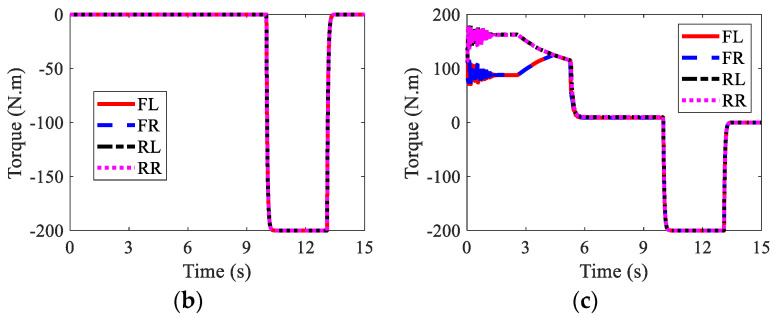
Wheel torques of AMR with mechanical braking in Case 1: (**a**) IWM; (**b**) EMB; (**c**) sum.

**Figure 12:**
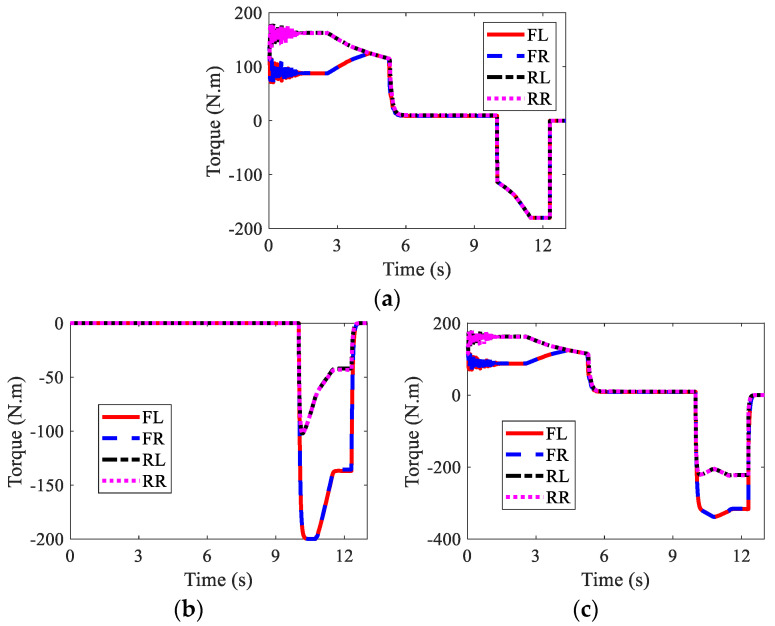
Wheel torques of AMR with hybrid braking in Case 1: (**a**) IWM; (**b**) EMB; (**c**) sum.

The wheel torques of AMR for three kinds of braking modes are displayed in Figure 10, Figure 11 and Figure 12, respectively. In the regenerative braking mode, only IWMs worked, in charge of both drive and control. In the mechanical braking mode, IWMs were only used for drive, and EMBs were used for braking. Therefore, the torques of IWMs changed to zero after 10th second. In the hybrid braking mode, both IWMs and EMBs were used for braking. EMBs could compensate the rest braking force for IWMs, shortening the braking time and braking distance.

From the above simulation results, it can be seen that the regenerative braking mode was beneficial to braking energy recovery. However, it led to longer braking distance, which reduces braking safety. The mechanical braking mode could shorten the braking distance but not recover the braking energy. In general, the hybrid braking mode had the advantages of the above two kinds of braking modes, i.e., maximizing the regenerative braking efficiency and advancing the braking safety.

### 5.2. Simulation Case 2

This case aimed to validate the AFTC algorithm for the AMR on a curved road; the hybrid braking mode was used. The AMR accelerated to 20 m/s and then started to brake after the 12th second. However, failure of the FL IWM occurred at the 10th second and failure of the RL IWM at the 12th second.

**Figure 13:**
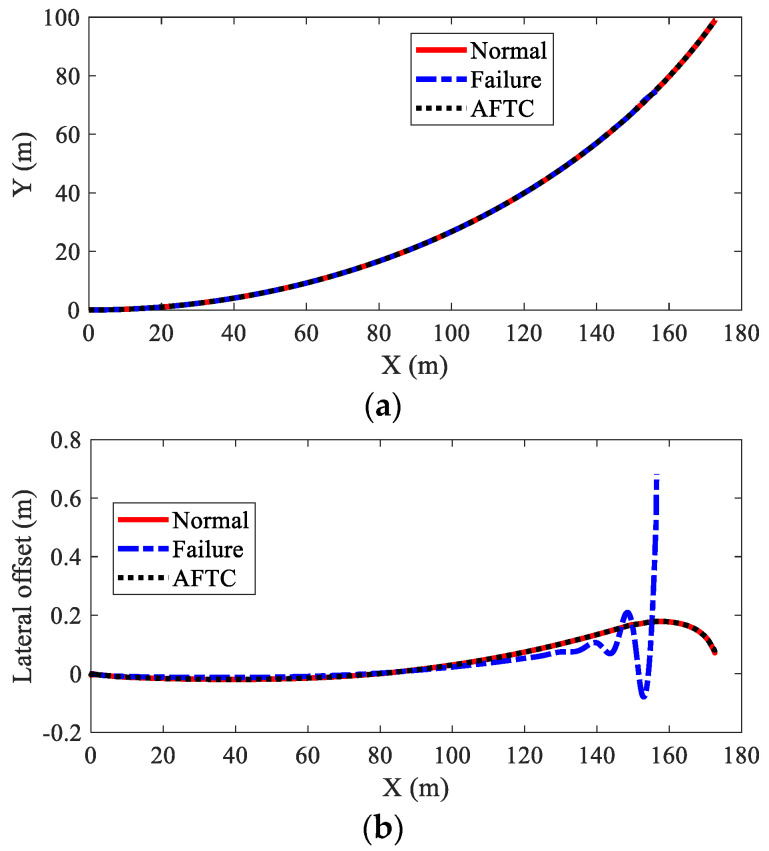
Path-tracking result of AMR in Case 2: (**a**) moving trajectories; (**b**) lateral offset.

**Figure 14:**
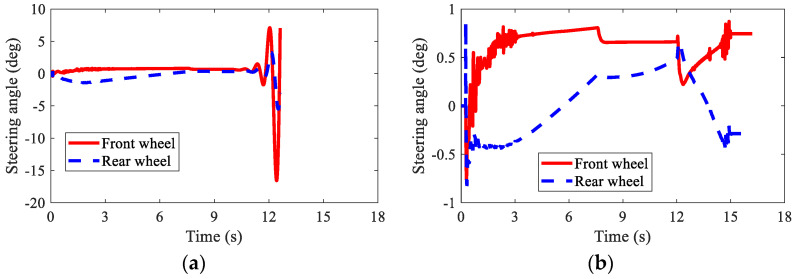
Steering angles of AMR in Case 2: (**a**) braking failure; (**b**) AFTC.

Figure 13 shows the path-tracking results of AMR under three kinds of conditions, i.e., normal (no failure), failure (without AFTC), and AFTC. It can be seen from Figure 13b that without AFTC, the AMR departed from its target path after braking failure, showing a large lateral offset. With AFTC, the AMR could realize lane-keeping after the braking failure and brake safely until stopped, as in the normal condition. The steering angles of AMR are illustrated in Figure 14. After the braking failure of IWMs, the AMR showed very large steering angles to realize lane-keeping when without AFTC. However, with AFTC, the AMR could use torque redistribution to guarantee brake safety and lateral stability.

**Figure 15:**
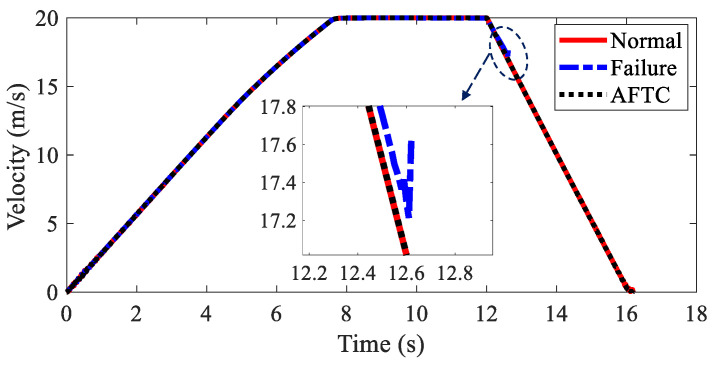
Velocity of AMR in Case 2.

The velocities of AMR under three kinds of conditions are depicted in Figure 15. Due to the loss of stability, the simulation was stopped at the 12.6 s when without AFTC. The AMR could not finish the braking process after the braking failure of the IWMs. With AFTC, the AMR could realize safe braking as in the normal condition.

**Figure 16:**
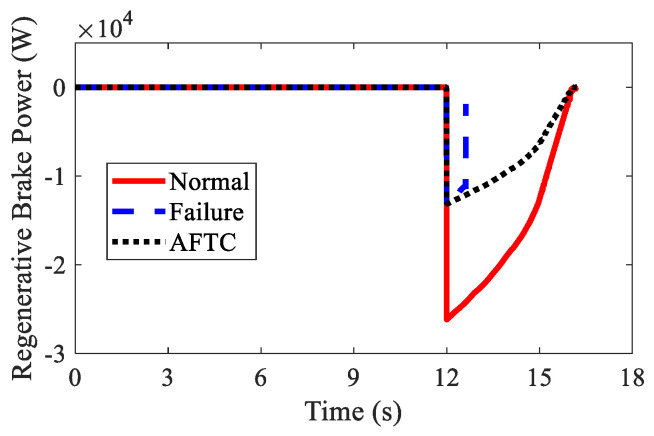
Regenerative braking power of AMR in Case 2.

The regenerative braking results are shown in Figure 16 and Table 3. In spite of the braking failure, the AFTC algorithm could help the AMR recover the braking energy up to $3.43\times 10^{4}$ J. Due to the braking failure of FL and RL IWMs, the recovered braking energy was smaller than in the normal condition.

**Figure 17:**
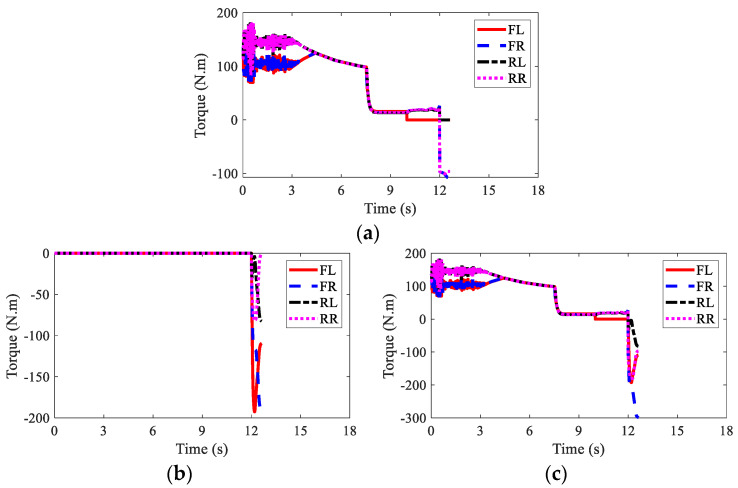
Wheel torques of AMR with failure in Case 2: (**a**) IWM; (**b**) EMB; (**c**) sum.

**Figure 18:**
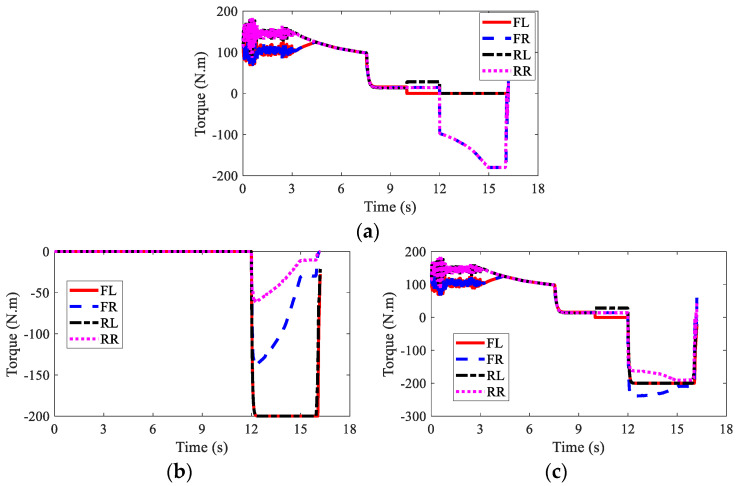
Wheel torques of AMR with AFTC in Case 2: (**a**) IWM; (**b**) EMB; (**c**) sum.

The wheel torques of AMR with failure and with AFTC are illustrated in Figure 17 and Figure 18, respectively. Due to the failure of FL and RL IWMs, the torques of the two IWMs changed to zero after the 10th second and the 12th second, respectively. Without AFTC, the AMR could not adjust its torque distribution to guarantee lateral stability. However, with AFTC, the EMBs redistributed the brake torque to compensate the braking force and overcome the external yaw moment caused by the braking failure of IWMs (Figure 18a,b).

### 5.3. Simulation Case 3

In this case, the braking failure condition of three IWMs was studied, further validating the effectiveness of the AFTC algorithm. The AMR accelerated to 20 m/s and then started to brake after the 12th second. However, the FL IWM had a failure at the 10th second, and the RL and RR IWMs had a braking failure at the 12th second.

**Figure 19:**
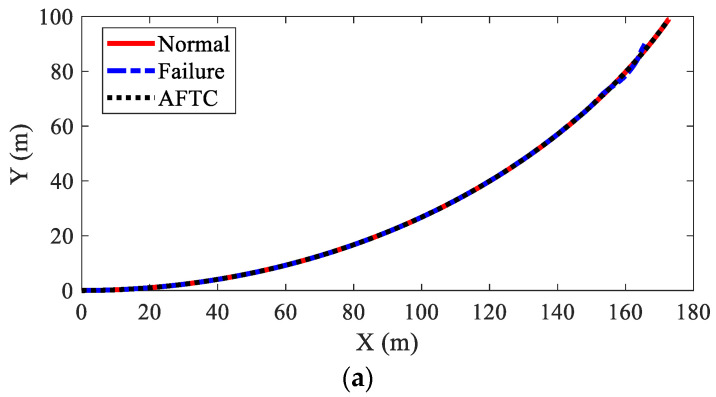

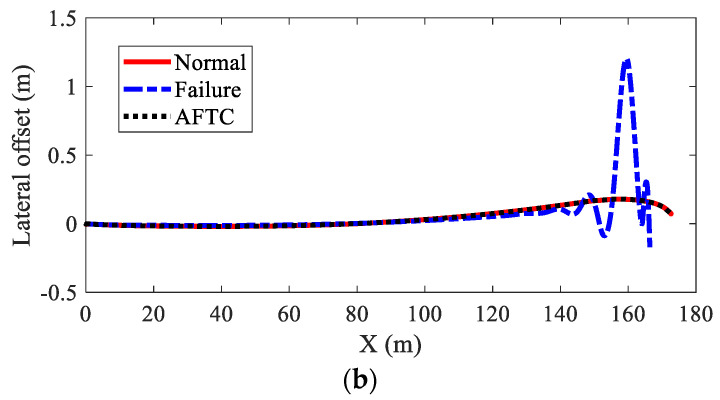
Path-tracking result of AMR in Case 3: (**a**) moving trajectories; (**b**) lateral offset.

**Figure 20:**
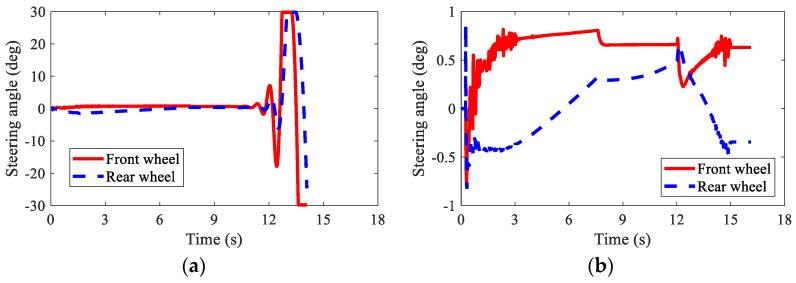
Steering angles of AMR in Case 3: (**a**) braking failure; (**b**) AFTC.

The path-tracking results of the AMR in this case are illustrated in Figure 19. This was similar to Case 2 in that without AFTC, the AMR departed from its original trajectory and lost stability after the braking failure of the IWMs. Moreover, the lateral offset was larger than that in Case 2. In spite of the increased failure numbers of IWMs, AFTC can help the AMR realize lane-keeping and safe braking. Figure 20 shows the steering angels of the AMR. It was found that the AMR had very large steering angles after the braking failure of IWMs, reaching the control boundaries. Despite this, the AMR could not guarantee stability and braking safety.

**Figure 21:**
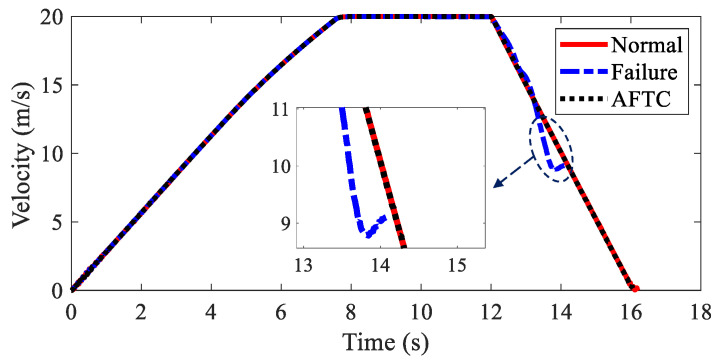
Velocity of AMR in Case 3.

Figure 21 shows the velocities of AMR under different conditions. Under the failure condition, the simulation was stopped at the 14.1 s due to the loss of stability of the AMR. However, the AFTC algorithm could help AMR address the braking failure of IWMs and finish the braking process safely.

**Figure 22:**
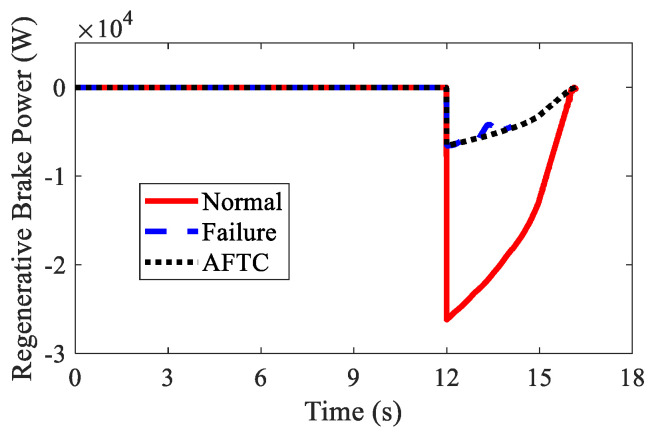
Regenerative braking power of AMR in Case 3.

The regenerative braking results of AMR are shown in Figure 22 and Table 4. In spite of the braking failure of three IWMs, the AFTC algorithm could help AMR recover braking energy up to −$1.72\times 10^{4}$ J using the normal IWM.

**Figure 23:**
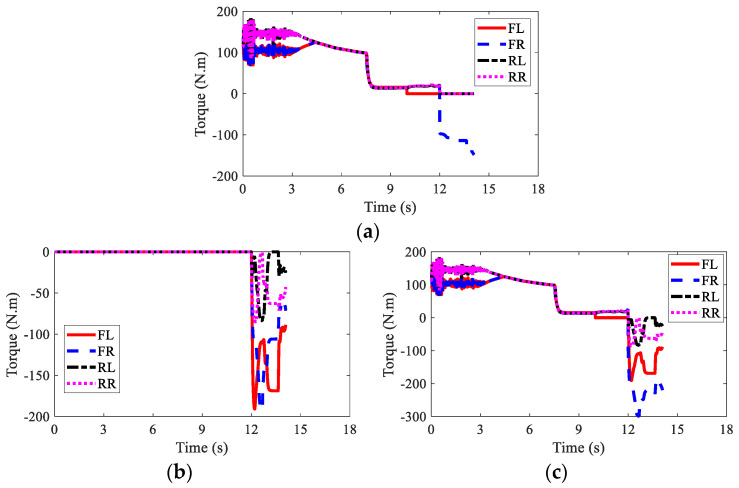
Wheel torques of AMR with failure in Case 3: (**a**) IWM; (**b**) EMB; (**c**) sum.

**Figure 24:**
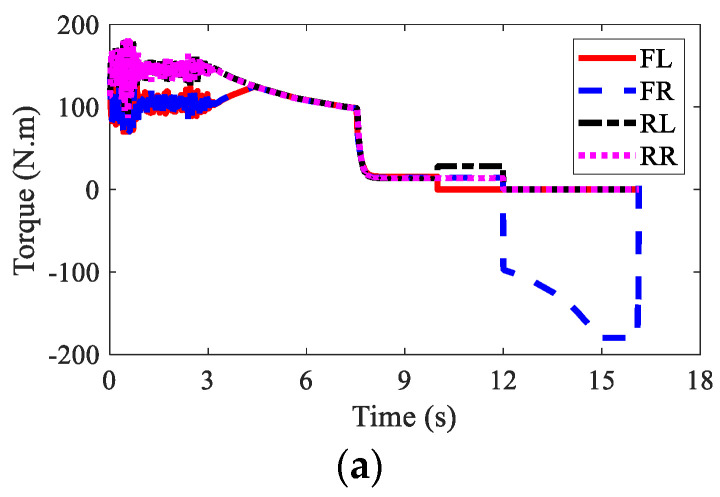

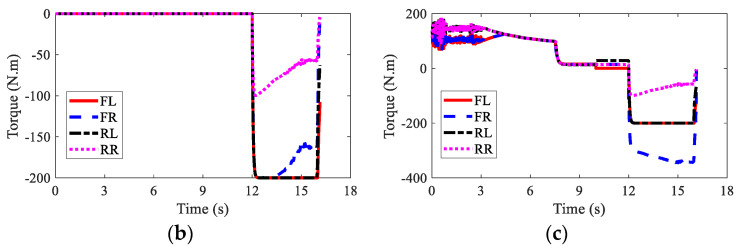
Wheel torques of AMR with AFTC in Case 3: (**a**) IWM; (**b**) EMB; (**c**) sum.

The wheel torques of AMR under the failure condition and the AFTC condition are displayed in Figure 23 and Figure 24, respectively. After the braking failure of three IWMs, the original torque distribution algorithm could not guarantee stability and braking safety. However, AFTC could help redistribute the torque of the normal IWM and four EMBs, recovering braking energy and guaranteeing braking safety and stability.

## 6. Conclusions

A chassis control framework was designed for an AMR. To address the braking failure of IWMs, an AFTC algorithm was studied by redistributing the braking torques of normal IWMs and four EMBs. Torque redistribution was carried out based on the WLS method. Three simulation cases were conducted to evaluate the feasibility and effectiveness of the proposed control algorithms. The simulation results indicate that the hybrid braking mode can help AMR recover the braking energy and advance braking safety. Moreover, the AFTC algorithm can deal with the braking failure of IWMs and realize braking energy recovery at the same time.

The hybrid conditions of IWM braking failure and EMB braking failure will be studied in future work.

## Figures and Tables

**Table 1:** AMR parameters for simulation.

Parameters	Value	Parameters	Value
m (kg)	431	$l_{f}$ (m)	0.829
$C_{D}$	0.28	$l_{r}$ (m)	0.705
A (m^2^)	0.97	l (m)	1.534
ρ (kg/m^3^)	1.2258	B (m)	0.97
$f_{r}$	0.008	$I_{w}$ (kg·m^2^)	0.67
$I_{z}$ (kg·m^2^)	217	$R_{w}$ (m)	0.298

**Table 2:** Comparative studies of three braking modes in Case 1.

	Regenerative Braking	Mechanical Braking	Hybrid Braking
**Braking distance (m)**	42.74	33.15	27.33
**Braking time (s)**	4.04	3.09	2.32
**Regenerative energy (J)**	$6.10\times 10^{4}$	0	$3.29\times 10^{4}$

**Table 3:** Regenerative braking energy of AMR in Case 2.

	Normal	Failure	AFTC
**Regenerative energy (J)**	$6.85\times 10^{4}$	$7.67\times 10^{3}$	$3.43\times 10^{4}$

**Table 4:** Regenerative braking energy of AMR in Case 3.

	Normal	Failure	AFTC
**Regenerative energy (J)**	$6.84\times 10^{4}$	$1.14\times 10^{4}$	$1.72\times 10^{4}$

## Data Availability

The data presented in this study are available on request from the corresponding author. The data are not publicly available due to privacy reason.
